# Laboratory Quantification of Gaseous Emission from Alternative Fuel Combustion: Implications for Cement Industry Decarbonization

**DOI:** 10.3390/ma18214859

**Published:** 2025-10-23

**Authors:** Ofelia Rivera Sasso, Elias Ramirez Espinoza, Caleb Carreño Gallardo, Jose Ernesto Ledezma Sillas, Alberto Diaz Diaz, Omar Farid Ojeda Farias, Carolina Prieto Gomez, Jose Martin Herrera Ramirez

**Affiliations:** 1 Centro de Investigacion en Materiales Avanzados, S.C. (CIMAV), Av. Miguel de Cervantes #120, Complejo Industrial Chihuahua, Chihuahua 31136, Mexico; ofelia.rivera@cimav.edu.mx (O.R.S.); elias.ramirez@cimav.edu.mx (E.R.E.); caleb.carreno@cimav.edu.mx (C.C.G.); jose.ledezma@cimav.edu.mx (J.E.L.S.); alberto.diaz@cimav.edu.mx (A.D.D.); 2 GCC, Vicente Suarez y Sexta s/n, Zona Industrial Nombre de Dios, Chihuahua 31105, Mexico; ofojeda@gcc.com

**Keywords:** waste-derived fuels, cement industry, emission factors, gaseous emissions, sustainable production

## Abstract

The cement industry accounts for approximately 7% of global CO_2_ emissions, with fuel combustion contributing 40% of sectoral emissions. Alternative fuels from industrial and municipal waste offer emission reduction opportunities while addressing waste management challenges. This study quantifies real-time gaseous emissions (CO_2_, CO, NO_x_, and SO_2_) from seven alternative fuels—sawdust (SD), pecan nutshell (PNS), wind blade waste (WBW), industrial hose waste (IHW), tire-derived fuel (TDF), plastic waste (PW), and automotive shredder residue (ASR)—during calcination at 850 °C. Bituminous coal served as the reference fuel. Gas concentrations were continuously monitored using the testo 350 portable gas analyzer. Emission factors were calculated on a mass basis (kg/kg fuel) and energy basis (kg/GJ) for standardized comparisons. Alternative fuels consistently produced lower CO_2_ emission factors than coal, with biomass-derived fuels (SD and PNS) showing reductions of 45% and 38%, respectively. Most alternative fuels generated lower CO and NO_x_ emissions per unit energy due to their higher volatile matter content, promoting complete combustion. TDF was an exception, exhibiting 2.8 times higher CO emissions. SO_2_ emissions were negligible except in the case of TDF (0.14% sulfur content). The measured emission factors were 15–30% lower than theoretical IPCC values, confirming the environmental viability of alternative fuels as coal substitutes in cement production.

## 1. Introduction

Gas emissions from the cement industry represent a major environmental concern, with the sector responsible for approximately 7% of global CO_2_ emissions. Of this proportion, around 40% originates from fuel combustion and energy use during cement production [1]. In an effort to reduce its carbon footprint as part of its sustainability strategy, the industry is increasingly turning to alternative fuels [2]. These fuels, often derived from industrial and municipal waste materials, offer a dual benefit: they support emission reduction efforts while simultaneously helping to divert waste from landfills [3]. The implementation of alternative fuels has already demonstrated meaningful environmental advantages, contributing to an estimated 12% reduction in CO_2_ emissions within the cement sector [2].

Currently, the cement industry primarily utilizes a variety of agricultural waste and industrial streams—such as biomass, used tires, rubber waste, and plastic waste—as sources of alternative fuels [4]. Beyond emission reduction potential, the thermal efficiency implications of alternative fuel adoption must be considered. Specific heat consumption for clinker production (typically 3.0–3.5 GJ/t clinker for modern coal-fired kilns) is influenced by fuel characteristics, particularly heating value and moisture content [5,6]. Alternative fuels with high heating values (>25 MJ/kg, comparable to bituminous coal at 25–30 MJ/kg) can maintain or improve thermal efficiency, while lower-energy fuels (<20 MJ/kg) may require increased mass feed rates to deliver equivalent thermal input [7]. Moisture content represents another critical parameter: fuels with >15% moisture impose energy penalties for water evaporation (2.26 MJ/kg H_2_O), potentially increasing specific heat consumption by 0.1–0.3 GJ/t clinker depending on substitution rate and moisture level [8]. Our previous characterization [9] showed that the alternative fuels examined here span a wide range of heating values (SD: 16.9 MJ/kg; PNS: 18.0 MJ/kg; TDF: 36.5 MJ/kg; PW: 29.0 MJ/kg), with correspondingly diverse thermal efficiency implications that must be balanced against their emission profiles.

However, the adoption of these fuels raises several critical questions: Do alternative fuels truly result in lower CO_2_ emissions compared to conventional fossil fuels? What types of gases are released during their combustion, and in what quantities? Addressing these questions is essential to fully understanding the environmental impact and feasibility of integrating alternative fuels into cement production.

In our previous study [9], we comprehensively analyzed the chemical composition of various alternative fuels—including sawdust (SD), pecan nutshell (PNS), wind blade waste (WBW), industrial hose waste (IHW), tire-derived fuel (TDF), plastic waste (PW), and automotive shredder residue (ASR)—focusing on key elements such as carbon, nitrogen, oxygen, hydrogen, and sulfur. The results revealed that SD, PNS, WBW, and ASR contained lower levels of carbon compared to the other fuels. Nitrogen was primarily present in WBW, IHW, TDF, PW, and ASR, indicating potential for NO_x_ formation during combustion. Sulfur, which contributes to SO_2_ emissions, was detected only in TDF, making it the sole sample with a significant sulfur content.

In addition to elemental analysis, thermogravimetric analysis coupled with mass spectrometry (TGA-MS) was used to study gas emissions during the calcination of these fuels at 850 °C [10]. The primary gases released were CO_2_, H_2_O, and CO, along with trace gases such as C_3_H_3_ and COOH. NO emissions were observed for WBW, IHW, TDF, PW, and ASR, aligning with their higher nitrogen content. SO_2_ emissions were detected exclusively from TDF, consistent with its sulfur composition. Importantly, none of the alternative fuels emitted hazardous compounds such as HCl, dioxins, and furans, indicating a cleaner combustion profile compared to certain traditional waste materials.

Despite these advances, there remains a significant lack of detailed information regarding the concentration of gas emissions from alternative fuels used in the cement industry. For instance, Shakya et al. [11] assessed emissions from tire waste using a UV–visible spectrophotometer and reported emission factors ranging from 21 µg/g to 49 µg/g for CO, 102 to 820 µg/g for SO_2_, and 3 µg/g to 9 µg/g for NO_2_. Similarly, Cereceda-Balic et al. [12] investigated emissions from wood combustion under controlled conditions, reporting emission factors of 39–49 g/kg for CO, 1701–1947 g/kg for CO_2_, 0.41–0.61 g/kg for NO_x_, and 0.37–0.48 g/kg for SO_2_.

Bhattacharya et al. [13] quantified CO_2_ and CO emission factors for wood and charcoal combustion in residential cookstoves using a gas analyzer. Their findings revealed distinct emission patterns between the fuel types: wood produced CO_2_ emission factors ranging from 1560 to 1620 g/kg and CO emission factors from 19 to 136 g/kg, while charcoal generated significantly higher emissions, with CO_2_ factors of 2155 to 2567 g/kg and CO factors of 35 to 198 g/kg. The substantially higher emission factors for charcoal reflect its elevated carbon content and different combustion characteristics compared to wood. Notably, the wide variability in CO emission factors (wood: 19–136 g/kg; charcoal: 35–198 g/kg) highlights the strong influence of combustion conditions and stove design on incomplete combustion products. These cookstove-based emission factors provide a useful baseline for comparisons, though the combustion temperatures and oxygen availability in residential applications differ significantly from the high-temperature industrial conditions (1450 °C) employed in cement production processes.

While the aforementioned studies provide valuable insights, they were primarily intended to evaluate the environmental impacts of waste incineration or disposal, rather than to characterize emissions from waste-derived materials used as alternative fuels in cement kilns.

Another commonly referenced source of emission data is the Intergovernmental Panel on Climate Change (IPCC), which publishes standardized emission factors that are frequently used by cement plants to evaluate the sustainability and environmental performance of various fuels. However, the IPCC emission factors have notable limitations when applied under real-world conditions. First, they are based on the assumption that all carbon in a fuel is fully oxidized to CO_2_ during combustion, which is not always the case in industrial settings, where incomplete combustion can occur. Second, these factors do not account for all types of alternative fuels, particularly those derived from plastic waste and other complex industrial residues. Additionally, emerging waste streams such as wind blade waste, increasingly available due to renewable energy infrastructure decommissioning, are not taken into account by the IPCC.

Larsen and Astrup [14] investigated CO_2_ emission factors from mixed municipal waste combustion by correlating emission rates with the carbon content of heterogeneous waste streams, including food waste, paper, glass, plastic, and metal components. Their analysis yielded emission factors ranging from 27 to 40 kg CO_2_/GJ, demonstrating the significant variability inherent in mixed waste materials due to compositional heterogeneity. The relatively wide emission factor range reflects the diverse carbon contents and combustion behaviors of different waste fractions: organic materials such as food waste and paper typically exhibit lower carbon densities and may have a substantial moisture content, while plastic components contribute higher carbon concentrations and heating values. This compositional variability in mixed waste streams presents challenges for accurate emission prediction in industrial applications, as the specific waste composition can fluctuate considerably depending on source, season, and local waste management practices. The energy-normalized approach employed by these authors provides a standardized framework for comparing diverse waste types, though the heterogeneous nature of mixed waste streams makes it difficult to establish consistent emission benchmarks for industrial fuel applications.

Many alternative fuels are categorized under broad labels such as “industrial waste,” which may not accurately reflect their specific chemical composition or combustion behavior [15,16]. As a result, emission estimates based on IPCC factors may lack precision and fail to represent the actual emissions observed in cement plants. This discrepancy underscores the need for more comprehensive, fuel-specific emission data to ensure accurate environmental assessments and support informed decision-making in alternative fuel adoption.

To address this knowledge gap, this study aims to evaluate the time-dependent concentrations and quantify the emissions of key gases—CO_2_, CO, NO_x_, and SO_2_—resulting from the combustion of various alternative fuels. To enable a standardized comparison, the emissions of each gas are normalized using the lower heating value (LHV) of the corresponding fuel. The waste materials analyzed include sawdust (SD), pecan nutshell (PNS), wind blade waste (WBW), industrial hose waste (IHW), tire-derived fuel (TDF), plastic waste (PW), and automotive shredder residue (ASR). Bituminous coal, a conventional fossil fuel commonly used in cement manufacturing, is included as a baseline for comparison.

By investigating the gaseous emissions—primarily governed by the intrinsic chemical compositions of the fuels—associated with these diverse waste materials, this study provides valuable insights into their environmental performance. Its findings will help determine the feasibility and sustainability of integrating alternative fuels into cement production processes.

## 2. Materials and Methods

### 2.1. Materials

The biomass and industrial waste materials utilized in this study were sourced from established agroindustries and industrial suppliers in the state of Chihuahua, Mexico, which generate these streams as consistent byproducts of their production processes. The samples included sawdust (SD), pecan nutshell (PNS), wind blade waste (WBW), industrial hose waste (IHW), tire-derived fuel (TDF), plastic waste (PW), and automotive shredder residue (ASR). Bituminous coal served as the reference fuel for comparative analysis. Representative samples were collected over a 6-month period to capture typical compositional characteristics, ensuring that the materials evaluated reflect the quality and variability of fuels available for industrial cement production. The chemical and physical properties of each fuel, including compositional stability verification, were comprehensively characterized and reported in our previous work [9].

These waste streams are supplied through long-term agreements with multiple regional industrial partners, ensuring continuous availability over multi-year operational horizons. Regional waste generation capacity substantially exceeds the plant’s consumption requirements, providing secure feedstock supply chains. The cement industry in Mexico has achieved alternative fuel substitution rates of 15–25% on a thermal energy basis, with continuous growth as waste management infrastructure expands [17].

### 2.2. Sample Preparation

The preparation methodology for characterization was adapted to the distinct physicochemical properties of each waste material. All samples underwent a standardized size reduction process using the SM 100 cutting mill (Retsch, Newtown, PA, USA) to yield a uniform particle size of 0.5 mm. After milling, the samples were subjected to controlled drying procedures and subsequently stored in desiccators to prevent moisture absorption and maintain integrity. For detailed information on the specific preparation techniques applied to each material type, refer to [9,10].

While detailed techno-economic analysis was beyond this study’s scope, literature values provide indicative preprocessing costs for waste-derived fuels. Mechanical size reduction typically requires 15–50 kWh/t depending on initial particle size and target fineness, with associated operational costs of $5–20/t for industrial-scale milling operations [18,19]. However, the economic viability of alternative fuels in cement production is significantly influenced by waste management responsibilities: in many jurisdictions, waste generators bear disposal costs, creating economic incentives for cement plants to accept these materials. When combined with avoided coal procurement costs ($60–120/t) and lower carbon tax exposure, alternative fuels often provide net economic benefits beyond their environmental advantages. Regional variations in waste management regulations, energy prices, and carbon pricing mechanisms significantly influence the economic attractiveness of specific alternative fuel strategies.

### 2.3. Experimental Setup and Methodology

#### 2.3.1. Combustion System Design

**Figure 1:**
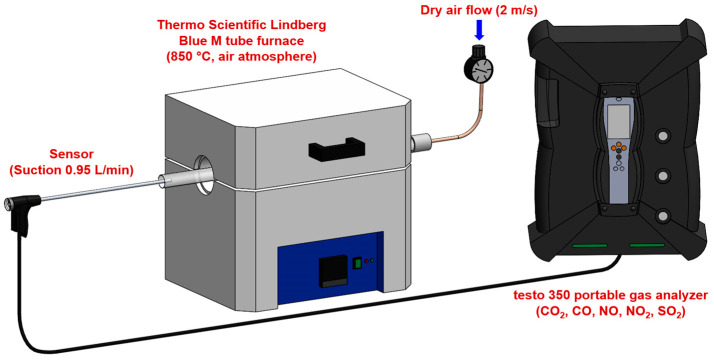
Experimental setup for gas concentration measurement during fuel combustion.

The experimental setup consisted of the Thermo Scientific Lindberg Blue M tube furnace (Thermo Scientific, Waltham, MA, USA) configured for controlled combustion analysis (Figure 1). The system had the following characteristics:

- Furnace specifications: a maximum operating temperature of 1200 °C with a temperature stability of ±5 °C, a heating rate of 10 °C/min to reach 850 °C, and isothermal hold capability for consistent combustion conditions. The temperature of 850 °C was selected to replicate the conditions in the precalciner zone of cement kilns, where alternative fuels are typically injected. At an industrial scale, the precalciner operates at temperatures between 800–900 °C, and alternative fuels introduced at this point undergo rapid combustion to provide heat for calcium carbonate decomposition [5]. This temperature represents the primary combustion environment for alternative fuels in modern cement production systems. The tube furnace’s circumferential heating elements create a uniform temperature profile within the isothermal zone (±5 °C), verified using a calibrated K-type thermocouple. Heat transfer to samples occurs primarily through radiation from furnace walls (dominant at 850 °C), supplemented by conduction and convection. Samples were consistently positioned at the center of the 15 cm isothermal zone to ensure identical thermal conditions across all experiments.
- Gas supply system: a dry air supply with flow velocity control at 2 m/s, flow rate monitoring using mass flow controllers, air composed of 21% O_2_ and 79% N_2_ (standard atmospheric composition), and an excess air (λ) ratio maintained at 1.5 to ensure complete combustion. The excess air ratio of λ = 1.5 was selected to represent typical industrial precalciner operating conditions (λ = 1.3–1.7) and to ensure adequate oxygen availability for complete combustion across all fuel types, while maintaining comparability between experiments.
- Literature data (Table 1) demonstrate that excess air ratio significantly influences combustion completeness and pollutant formation, though specific responses vary by fuel type and combustion system. For CO emissions, insufficient excess air (λ < 1.3) consistently results in elevated concentrations due to incomplete oxidation, while λ values above 1.5 typically provide diminishing returns in CO reduction [20,21]. NO_x_ formation exhibits more complex behavior: at moderate temperatures (850 °C), fuel-NO_x_ dominates over thermal-NO_x_, with emissions generally increasing 15–40% as λ increases from 1.3 to 1.8 due to enhanced oxidative conditions for nitrogen conversion [22,23,24]. The selected value of λ = 1.5 represents a practical compromise that ensures complete combustion (minimizing CO) while avoiding excessive oxygen that could exacerbate NO_x_ formation, particularly for nitrogen-rich fuels. Systematic parametric investigation of λ effects for each alternative fuel would provide valuable optimization guidance but was beyond this study’s scope, which focused on establishing baseline emission factors under standardized, industrially relevant conditions for direct fuel comparison.

- Sample handling: quartz boats for sample containment during combustion, a sample mass of 1.000 ± 0.001 g for all experiments, and a positioning system for consistent sample placement within the furnace.

While this laboratory configuration provides controlled, reproducible conditions for fundamental fuel characterization, industrial cement kilns operate under different conditions that may affect absolute emission magnitudes. Literature comparisons between laboratory and industrial-scale combustion indicate that CO_2_ emission factors typically agree within ±15–30%, as CO_2_ production is primarily governed by fuel carbon content [25,26]. However, combustion-sensitive species such as CO and NO_x_ may exhibit larger variations (±30–60%) between laboratory and industrial measurements due to differences in turbulent mixing, temperature gradients, and residence time distributions [7,27]. Industrial kilns generally achieve more complete combustion through longer effective residence times (minutes vs. seconds), higher peak temperatures in the burning zone (1400–1800 °C), and intense turbulent mixing, which can reduce CO emissions but potentially increase thermal NO_x_ formation relative to controlled laboratory conditions [5,8]. The controlled environment employed here enables isolation of fuel-specific emission characteristics under reproducible conditions, providing baseline data for fuel comparison, screening, and selection—with the understanding that industrial implementation requires validation under full-scale operating conditions.

#### 2.3.2. Gas Concentration Measurement

All experiments were conducted at ambient pressure and temperature (~101.3 kPa, 22 ± 2 °C). Gas concentrations were measured using the testo 350 portable gas analyzer (Testo, West Chester, PA, USA) with the following specifications:

- Measurement parameters: CO_2_ (0–50% vol., resolution 0.01% (0–25 vol.%) and 0.1 vol.% (>25 vol.%)), CO (0–10,000 ppm, resolution 1 ppm), NO (0–4000 ppm, resolution 1 ppm), NO_2_ (0–500 ppm, resolution 0.1 ppm), and SO_2_ (0–5000 ppm, resolution 1 ppm). Note that NO_x_ measurements are the sum of individual NO and NO_2_ measurements.
- Data acquisition: a sampling interval of 1 s, response time t_90_ < 40 s for all measured gases, continuous monitoring throughout the entire combustion process, and real-time data logging for subsequent analysis. The sampling interval of 1 s substantially exceeds the Nyquist criterion relative to the instrument response time (t_90_ < 40 s) and observed combustion time scales (60–960 s), ensuring accurate representation of concentration profiles without aliasing artifacts.

#### 2.3.3. Experimental Protocol

The experimental procedure followed a standardized protocol. Prior to sample introduction, the system was purged with dry air for 10 min. Each sample was then placed in the furnace, preheated to 850 °C. Gas concentrations were continuously monitored from the moment of sample introduction until they returned to baseline levels.

All experiments were performed in triplicate (n = 3) for each fuel type to quantify experimental variability and ensure reproducibility. Each replicate consisted of an independent combustion test with a fresh sample (1.000 ± 0.001 g) under identical conditions. Reported emission factors represent mean values from three replicates, with uncertainties expressed as standard deviations.

#### 2.3.4. Data Processing and Analysis

Data processing and emission factor calculations were performed as follows:

- Gas concentration profiles: raw concentration data (ppm) recorded as a function of time, baseline correction applied to account for background concentrations, and peaks identified and integrated using Simpson’s rule [28].
- Mass-based emission factors (kg gas/kg fuel): calculated using Equation (1) [15].

(1) $${EF}_{mass}=\frac{\int_{0}^{t}C\left(t\right)Q\rho dt}{m_{fuel}}$$

where *C*(*t*) = gas concentration at time *t* (ppm); *Q* = gas flow rate (m^3^/s); *ρ* = gas density (kg/m^3^); and *m_fuel_* = fuel mass (kg).

- Energy-based emission factors (kg gas/GJ): calculated using Equation (2) [15].

(2) $${EF}_{energy}=\frac{{EF}_{mass}}{LHV}$$

where *LHV* = lower heating value of the fuel (MJ/kg).

#### 2.3.5. Lower Heating Value Determination

The lower heating value (LHV) for each fuel was determined using bomb calorimetry following the ASTM D5865/D5865M standard, as detailed in [9,29]. These data were essential for normalizing emission factors to energy content, allowing for meaningful comparisons between fuels with different heating values.

#### 2.3.6. Validation and Calibration

System validation and calibration procedures included the following:

- System validation: the system validation was performed using bituminous coal as a reference fuel. The measured CO_2_ emission factor for coal (18.5 kg CO_2_/GJ) falls within the literature range for bituminous coal (17.5–20.2 kg CO_2_/GJ), and the CO emission factor (71.5 g/kg) is consistent with published values for similar combustion temperatures [27]. Carbon mass balance calculations verified that measured CO_2_ and CO emissions accounted for the fuel carbon content within ±8% for all samples, confirming complete carbon accounting. Gas species identification was consistent with our previous TGA-MS analysis [10], providing independent analytical validation of the measurement system.
- Calibration procedures: the gas analyzer was calibrated daily using NIST-traceable certified span gases, with zero calibration performed using nitrogen. Regular maintenance and calibration checks were conducted according to the manufacturer’s specifications. Measurement reliability was ensured through these routine calibrations, triplicate testing (n = 3 per fuel), complementary TGA-MS analysis [10], and carbon mass balance verification (±8%). This integrated approach minimized systematic measurement errors and validated the reported emission factors.

## 3. Results

This section presents a comprehensive analysis of the temporal emission profiles and quantitative assessment of gaseous emissions from the analyzed alternative fuels during calcination at 850 °C. It first examines the time-dependent concentration patterns of each gas species (CO_2_, CO, NO_x_, and SO_2_), followed by quantitative emission factor calculations expressed both per unit mass and per unit energy. This approach enables a thorough understanding of the combustion behavior and environmental performance of each fuel relative to bituminous coal, the reference standard, providing essential data for industrial decision-making and regulatory compliance in cement production applications.

### 3.1. Time-Dependent Concentration of Gaseous Emissions

#### 3.1.1. Carbon Dioxide Emissions

**Figure 2:**
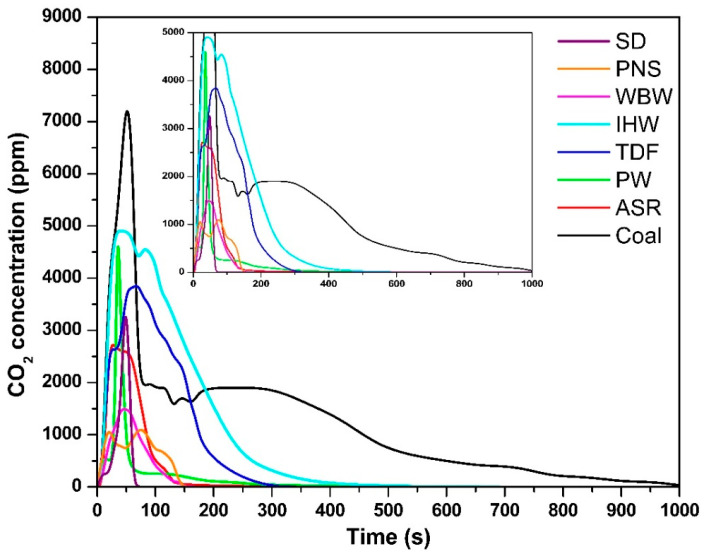
CO_2_ concentration as a function of time.

The temporal evolution of CO_2_ concentrations for all tested fuels is shown in Figure 2. The alternative fuels exhibited maximum CO_2_ concentrations of 3280 ppm (SD, 47 s), 1046 ppm (PNS, 22 s), 1468 ppm (WBW, 45 s), 4854 ppm (IHW, 53 s), 3795 ppm (TDF, 62 s), 4478 ppm (PW, 37 s), and 2713 ppm (ASR, 28 s), compared to coal’s 7158 ppm (51 s). A distinct combustion pattern emerged among the biomass- and plastic-based materials (SD, PNS, WBW, PW, and ASR), which demonstrated accelerated CO_2_ release kinetics. This behavior is primarily attributed to their elevated volatile matter content, which enhances fuel reactivity and promotes rapid thermal decomposition upon exposure to high temperatures [9]. These observations align with those of Ozer et al. [30], who demonstrated that a high volatile matter content facilitates faster CO_2_ evolution due to the preferential oxidation of light organic compounds containing carbon, hydrogen, and oxygen fragments [31].

In contrast, fuels with a higher fixed carbon content—specifically IHW, TDF, and coal—exhibited delayed CO_2_ release profiles. The presence of more stable carbon–carbon bonds in these materials reduces oxygen reactivity, consequently prolonging the oxidation process and extending the time required to reach peak CO_2_ concentrations [32]. This structural characteristic necessitates additional thermal energy and residence time to achieve complete carbon oxidation, consistent with the reported combustion behavior of similar carbonaceous materials [33,34]. The superior combustion performance of coal, evidenced by its highest CO_2_ concentration, reflects its elevated fixed carbon content and the consequent requirement for extended oxidation periods to break down these stable molecular structures [27,35].

The combustion durations varied significantly among the tested fuels: SD—60 s; PNS—140 s; WBW—140 s; IHW—360 s; TDF—240 s; PW—180 s; ASR—130 s; and coal—960 s. Alternative fuels consistently demonstrated shorter combustion periods compared to coal, primarily due to their higher volatile matter content. Volatile compounds, consisting of readily vaporized organic fragments, exhibit enhanced reactivity and undergo rapid oxidation to form CO_2_ or CO [36]. This elevated reactivity contributes to reduced ignition temperatures and accelerated thermal decomposition rates. Among the tested samples, SD demonstrated the shortest combustion duration, indicating superior ignition characteristics, attributable to their high concentration of reactive organic volatile compounds. This behavior is consistent with findings from the literature suggesting that volatile-rich fuels undergo rapid devolatilization processes, particularly under oxygen-limited conditions [36,37], which can promote incomplete combustion and the formation of intermediate products such as CO [27,31,38]. The extended combustion duration of WBW (140 s) despite its low carbon content (31.3%) is attributed to its complex fiber-reinforced composite structure and exceptionally high ash content (53% [9]). The inert ash acts as a diluent and forms surface layers that impede oxygen diffusion and reduce heat transfer rates to unreacted fuel. Additionally, carbon in WBW exists primarily in thermally stable aromatic polymer structures and fiber reinforcement, requiring longer residence times for complete oxidation compared to simple organic compounds in biomass fuels [9,10]. This contrasts with sawdust (45.5% C, 60 s combustion, 1.4% ash), which enables rapid, unobstructed combustion.

#### 3.1.2. Carbon Monoxide Emissions

**Figure 3:**
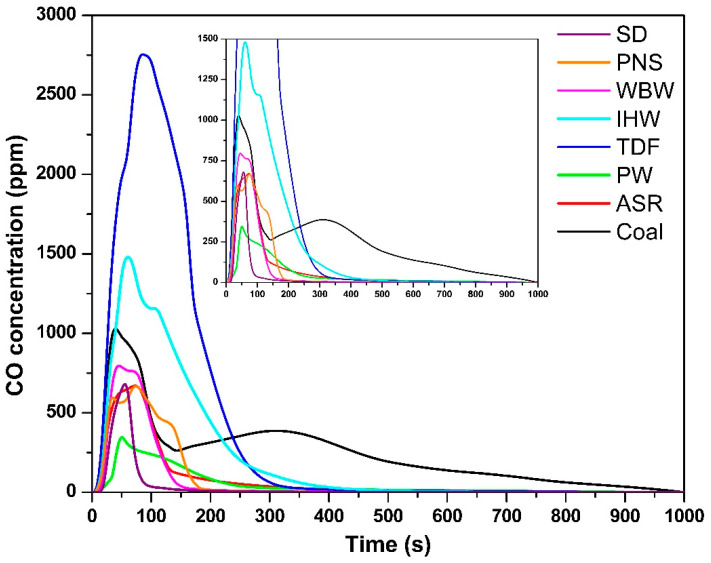
CO concentration as a function of time.

The temporal CO concentration profiles (Figure 3) followed similar trends to those observed for CO_2_ emissions. The maximum CO concentration for each fuel was as follows: SD—647 ppm, 55 s; PNS—666 ppm, 73 s; WBW—790 ppm, 45 s; IHW—1466 ppm, 60 s; TDF—2752 ppm, 88 s; PW—332 ppm, 50 s; ASR—670 ppm, 70 s; and coal—980 ppm, 37 s. Notably, IHW and TDF generated the highest CO emissions, substantially exceeding those of other fuels, including coal. This elevated CO production is attributed to their complex chemical composition, which includes synthetic polymers, rubber compounds, heavy hydrocarbons, and various chemical additives [9]. These components hinder complete combustion and promote the formation of CO as an intermediate oxidation product, particularly during the initial combustion phases [39]. This finding is consistent with those of Carmo-Calado et al. [40], who reported that structurally complex fuels tend to produce elevated CO levels due to incomplete combustion processes. Despite the provision of excess oxygen in the experimental setup, CO formation represents a typical intermediate step in the combustion process that cannot be completely eliminated [32].

From a thermodynamic perspective, CO is an obligate intermediate in carbon oxidation. The Boudouard equilibrium (C + CO_2_ ⇌ 2CO) shifts toward CO at 850 °C, particularly in carbon-rich zones [41]. The elevated CO emissions from TDF and IHW reflect both thermodynamic factors (high temperature, complex aromatic structures) and kinetic limitations (slow oxidation rates of dense carbonaceous structures, creating localized oxygen depletion). Complete oxidation to CO_2_ requires adequate oxygen supply and residence time; without these conditions, some CO inevitably escapes unreacted.

The CO emission durations paralleled those observed for CO_2_: SD—100 s; PNS—180 s; WBW—160 s; IHW—400 s; TDF—350 s; PW—300 s; ASR—300 s; and coal—960 s. This temporal correspondence reflects the progressive nature of combustion and oxidation processes. Fuels with a high volatile matter content—particularly SD, PNS, and WBW—exhibited shorter CO emission periods due to rapid ignition and early-stage volatile oxidation, which facilitates the transition from CO to CO_2_ [42]. In contrast, IHW, TDF, and coal demonstrated extended combustion periods, driven by their elevated fixed carbon content and complex molecular architectures. This behavior aligns with previous investigations of tire waste calcination at temperatures between 650 °C and 900 °C [43] and coal combustion between 500 °C and 700 °C [27]. These materials undergo slower thermal degradation, thereby delaying the oxidation of intermediate species such as CO [42]. Even under oxygen-rich conditions, their compact carbon–carbon bonding structure and reduced reactivity impede complete combustion, resulting in prolonged CO presence in exhaust streams [44]. This observation underscores the fundamental distinction between volatile-rich materials (biomass and certain plastics), which undergo rapid combustion, and denser, carbon-rich fuels (coal and TDF), which require extended time and energy for complete oxidation [45].

The exceptionally elevated CO emissions from TDF (2.8 times higher than coal) are directly attributable to its complex, multi-component chemical composition. TDF contains natural and synthetic rubber polymers (45–60%), carbon black filler (20–30%), and steel reinforcement (10–15%) [9,46]. During combustion, rapid thermal decomposition of rubber generates hydrocarbon radicals and aromatic compounds that undergo incomplete oxidation, producing CO as an intermediate. The carbon black phase—consisting of graphitic microstructures with high surface area—oxidizes slowly at 850 °C, with surface chemistry favoring CO desorption over complete oxidation to CO_2_. Additionally, the heterogeneous particle structure creates non-uniform temperature and oxygen distributions, with localized oxygen-depleted zones where CO formation is thermodynamically favored [47]. The multi-phase combustion process (volatile release → polymer decomposition → carbon black oxidation) extends over 350 s, during which substantial CO escapes before complete oxidation can occur despite excess air availability (λ = 1.5) [48]. This behavior contrasts with coal’s more homogeneous structure and combustion characteristics.

#### 3.1.3. Nitrogen Oxide Emissions

**Figure 4:**
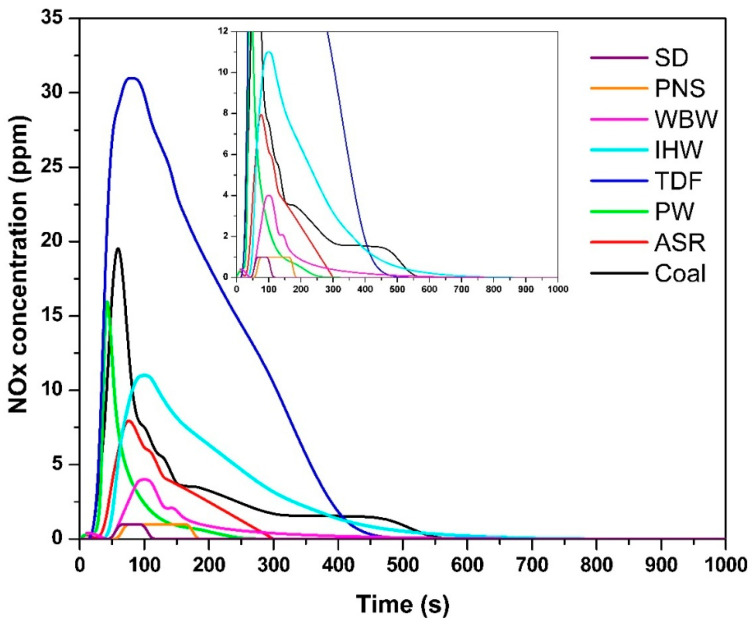
NO_x_ concentration as a function of time.

The temporal NO_x_ concentration profiles (Figure 4) demonstrated fuel-specific emission patterns, with the following maximum concentrations: SD—1 ppm, 68 s; PNS—1 ppm, 70 s; WBW—4 ppm, 100 s; IHW—11 ppm, 100 s; TDF—31 ppm, 80 s; PW—16 ppm, 42 s; ASR—8 ppm, 78 s; and coal—19 ppm, 60 s. The minimal NO_x_ concentrations observed for SD and PNS correspond directly to their low nitrogen content [9]. NO_x_ formation during combustion is governed by several factors, including a fuel’s nitrogen content, combustion temperature, and oxygen availability. Fuel-bound nitrogen reacts with oxygen at elevated temperatures to form NO and, to a lesser extent, NO_2_, with their sum reported as NO_x_ [49,50].

The minimal NO_x_ concentrations observed for SD and PNS (1 ppm) are at the lower resolution limit of the measurement system (resolution: 1 ppm NO). While these values represent detectable concentrations, the instrument accuracy specification (±5 ppm) exceeds the measured values, indicating that these should be interpreted as ‘trace levels below reliable quantification limits’ rather than precise quantitative measurements. Nevertheless, these low concentrations are fully consistent with the negligible nitrogen content of biomass fuels (<0.5% N [9]) and align with theoretical expectations and literature values for wood combustion NO_x_ emissions (0.4–0.6 g/kg [12]). The clear separation from nitrogen-rich fuels (IHW: 11 ppm, coal: 19 ppm, TDF: 31 ppm) confirms that the measurements capture meaningful differences in NO_x_ formation behavior.

Materials containing higher nitrogen concentrations—specifically TDF, PW, ASR, and coal—produced the most significant NO_x_ emissions. This correlation reflects the presence of nitrogen-rich compounds in synthetic materials (such as rubber and polymer components in TDF) and fossil-based fuels (coal), where organic nitrogen compounds are prevalent [27]. During thermal decomposition and combustion, these nitrogen-containing compounds undergo conversion to NO_x_ species over extended periods [51].

The NO_x_ emission durations were as follows: SD—110 s; PNS—180 s; WBW—180 s; IHW—400 s; TDF—400 s; PW—240 s; ASR—300 s; and coal—540 s. These values correlate with both the nitrogen content and the molecular complexity of nitrogen-containing compounds present in each fuel. More complex nitrogen-bearing molecular structures require longer residence times and higher temperatures to achieve complete oxidation, thereby extending NO_x_ formation periods [50]. TDF and coal, which both contain elevated nitrogen levels and exhibit prolonged combustion characteristics, demonstrated the longest NO_x_ emission periods. This behavior is consistent with the findings of Vicente et al. [27], who noted that a higher nitrogen content in fuel directly correlates with increased NO_x_ emissions. Therefore, the quantity and chemical speciation of nitrogen compounds, combined with combustion temperature and oxygen availability, play critical roles in determining both NO_x_ peak concentrations and emission duration.

#### 3.1.4. Sulfur Dioxide Emissions

**Figure 5:**
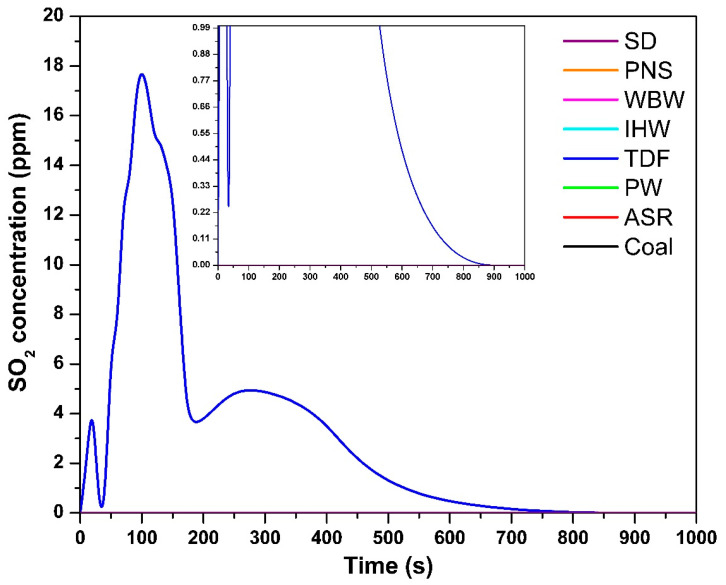
SO_2_ concentration as a function of time.

The temporal SO_2_ concentration profile in Figure 5 shows that, among all the tested fuels, only TDF produced measurable SO_2_ emissions, correlating with its sulfur content of 0.14% [9]. During combustion, fuel-bound sulfur undergoes oxidation to form SO_2_ under adequate oxygen availability and high-temperature conditions [27]. The maximum SO_2_ concentration for TDF reached 18 ppm at 100 s, indicating the onset of active sulfur oxidation. SO_2_ emissions persisted for approximately 500 s, suggesting the progressive release and oxidation of sulfur compounds throughout the combustion process. These findings are consistent with those of Shakya et al. [11], who reported detectable SO_2_ emissions from vehicle tire combustion proportional to sulfur content. The remaining samples exhibited negligible or undetectable SO_2_ emissions, corresponding to their minimal or absent sulfur content [9]. Without significant sulfur presence, there is insufficient substrate for SO_2_ formation during combustion.

The gas analyzer has an SO_2_ detection limit of 1 ppm (resolution: 1 ppm, accuracy: ±5 ppm). For all fuels except TDF, SO_2_ concentrations remained below this detection threshold throughout the combustion process. This is consistent with elemental analysis showing negligible sulfur content (<0.01%) in SD, PNS, WBW, IHW, PW, and ASR [9]. At sulfur levels below 0.01%, theoretical SO_2_ emissions would not exceed 0.2 mg/g fuel, corresponding to peak concentrations ≪ 1 ppm—well below the analyzer’s quantification capability. The absence of detectable SO_2_ therefore reflects genuinely low sulfur content rather than analytical limitations. Only TDF, with significantly higher sulfur content (0.14%), produced measurable SO_2_ emissions (maximum: 18 ppm, duration: ~500 s). The negligible SO_2_ emissions from most alternative fuels represent a clear environmental advantage, as SO_2_ contributes to acid rain formation and respiratory health impacts.

Sulfur in TDF exists primarily as elemental sulfur (from vulcanization), polysulfide crosslinks in rubber polymer chains, and organic sulfur compounds from vulcanization accelerators [9]. During combustion at 850 °C with excess air (λ = 1.5), these species undergo thermal decomposition and rapid oxidation to form SO_2_ as the dominant sulfur emission product. Our previous TGA-MS analysis [10] confirmed SO_2_ as the primary sulfur-containing species, with no detection of H_2_S or other reduced sulfur compounds under oxidizing conditions. A sulfur mass balance calculation shows approximately 56% recovery of fuel-bound sulfur as gaseous SO_2_, with the remainder likely retained in ash as sulfates (ZnSO_4_) or adsorbed on surfaces. The conversion efficiency of fuel sulfur to SO_2_ is typical for combustion under oxidizing conditions and confirms that the measured SO_2_ emissions represent the majority of environmentally relevant sulfur releases.

### 3.2. Mass-Based Emission Factors

**Figure 6:**
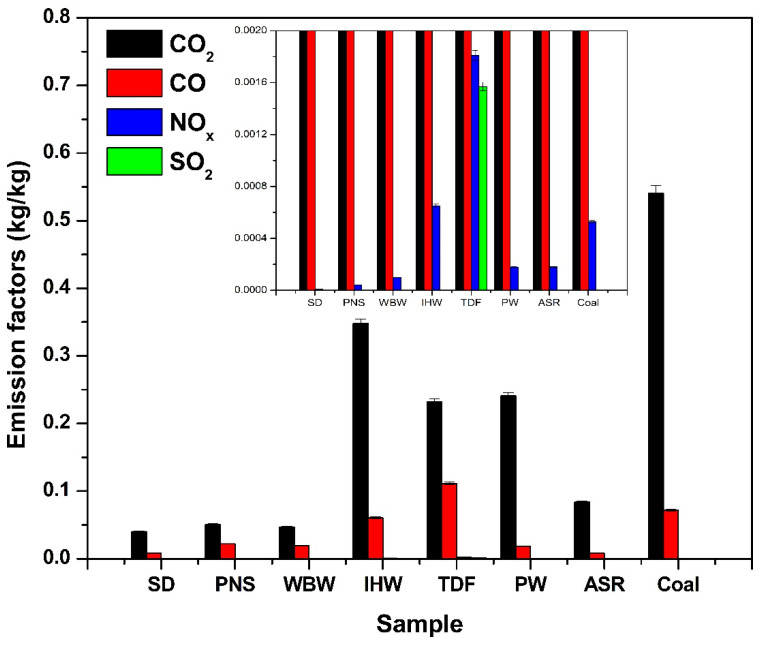
Mass-based emission factors for CO_2_, CO, NO_x_, and SO_2_ from alternative fuels compared to coal.

All emission data, including mean values and standard deviations for each emission factor (kg/kg), are presented in Figure 6 and Table 2. The quantitative analysis of gaseous emissions per kilogram of fuel (Figure 6) reveals that alternative fuels consistently produce lower CO_2_ emissions compared to coal, primarily due to their reduced carbon content. Since CO_2_ formation results from complete combustion of carbon-bearing compounds, fuels with lower carbon concentrations naturally generate proportionally less CO_2_. Coal’s elevated fixed carbon fraction leads to higher CO_2_ emissions upon combustion. Additionally, alternative fuels typically contain more volatile matter and oxygenated compounds, which can contribute to reducing net CO_2_ production [52]. Consequently, the lower carbon intensity and distinct compositional characteristics of alternative fuels result in decreased CO_2_ emissions per unit mass combusted [53].

SD, PNS, WBW, and ASR produced the lowest CO_2_ emissions among the tested samples, with SD exhibiting the minimum value. Quantitatively, SD and PNS achieved CO_2_ emission reductions of 45% and 38% compared to coal, respectively. This outcome directly correlates with their respective carbon contents: 45.5%, 50.8%, 31.3%, and 49.1% [9]. Conversely, IHW, TDF, and PW generated the highest CO_2_ emissions, consistent with their significantly elevated carbon contents of 82.9%, 90.0%, and 77.4%, respectively [9]. As CO_2_ represents the primary product of carbon oxidation during combustion, its emission is directly proportional to a fuel’s carbon concentration [25]. Therefore, the variation in CO_2_ emissions among the alternative fuels can be attributed primarily to differences in their carbon content.

CO emissions from alternative fuels were generally lower than those from coal, with TDF being the notable exception. This behavior reflects the elemental composition differences between fuel types. Most alternative fuels exhibit lower carbon density and higher hydrogen and oxygen content compared to coal [9]. These compositional characteristics promote more complete oxidation during combustion, reducing formation of CO as an incomplete combustion product. In contrast, TDF and coal possess a higher fixed carbon content and less volatile matter; thus, under limited oxygen availability or suboptimal combustion conditions, they tend to produce elevated CO levels. This occurs because fixed carbon requires higher temperatures and sustained combustion for complete oxidation, while insufficient oxygen or residence time results in partial oxidation, yielding CO rather than CO_2_ [54].

While both biomass (SD and PNS) and plastic-derived fuels (WBW, IHW, TDF, PW and ASR) are being explored as alternative solid fuels, their chemical composition and molecular structure result in distinct combustion behaviors and emission profiles. Biomass compounds, primarily composed of oxygenated biopolymers such as cellulose, hemicellulose, and lignin, contain high levels of oxygen and nitrogen in naturally bound forms. This composition promotes more complete oxidation, leading to lower CO emissions and shorter combustion times.

In contrast, plastic-derived fuels, such as those based on polyethylene, polypropylene, and polystyrene, consist mainly of highly reduced hydrocarbon chains with negligible oxygen content. These materials generally exhibit higher heating values, but their combustion is often less efficient due to the lack of oxygenated functional groups, resulting in higher CO and soot emissions under sub-stoichiometric conditions. Additionally, plastics that contain nitrogen-based functional groups (e.g., amines or urethanes) can contribute to NO_x_ formation, depending on combustion temperature and residence time. Overall, the low oxygen content and more stable molecular structures of plastics lead to slower burnout rates and increased potential for incomplete combustion compared to biomass. Detailed chemical speciation and thermal decomposition pathways for these fuels were characterized in our previous work using FTIR spectroscopy and TGA-MS [9,10], which identified the major functional groups and volatile products responsible for the emission patterns observed in this study.

NO_x_ emissions from alternative fuels were generally lower than those from coal, with the exception of IHW and TDF. This pattern primarily reflects the reduced nitrogen content in most alternative fuels. Materials such as SD, PNS, WBW, PW, and ASR contain less nitrogen compared to coal [9], limiting NO_x_ formation during combustion. Conversely, IHW and TDF may contain elevated concentrations of nitrogen-rich compounds, leading to increased NO_x_ emissions [9,10,55]. Similar results have been reported for NO_x_ emissions from tire combustion, ranging between 1.97 g and 2.9 g NO_x_ per kilogram when carried out at temperatures between 650 °C and 900 °C [43].

The SO_2_ emissions observed during TDF combustion align with values from the literature. The emission factor obtained in this study is consistent with continuous field measurements during landfill fires involving tire waste, which report SO_2_ emission factors ranging from 0.0008 g to 0.007 g SO_2_ per gram of tire burned. These variations reflect differences in combustion conditions and tire composition observed in real-world scenarios [11,56].

### 3.3. Energy-Based Emission Factors

**Figure 7:**
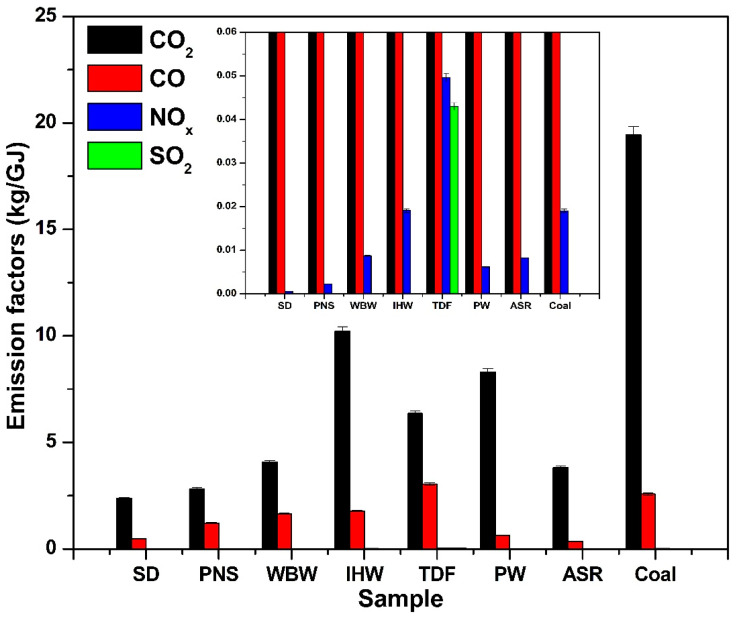
Energy-based emission factors for CO_2_, CO, NO_x_, and SO_2_ from alternative fuels compared to coal.

Normalization of emissions using the lower heating value (LHV) provides a standardized basis for fuel comparison, with associated standard deviations shown in Figure 7 and Table 2. Alternative fuels release less CO_2_ per gigajoule (GJ) of energy compared to coal, following the trend observed for mass-based emissions. The emission factors (kg CO_2_/GJ) for SD, PNS, WBW, and ASR are relatively low, reflecting their reduced carbon content compared to other samples. SD and PNS demonstrated the most significant reductions, with 45% and 38% lower CO_2_ emissions compared to coal, respectively. When compared to the IPCC-established range of emission factors for biomass (23.1–32.0 kg CO_2_/GJ) [15], the values obtained for SD and PNS in this study are lower.

IHW, TDF, and PW exhibited higher emission factors (kg CO_2_/GJ), aligning with their elevated carbon content; however, these values are nonetheless lower than the IPCC’s emission factors for industrial waste (30.0–50.0 kg CO_2_/GJ) [15]. The measured CO_2_ emission factors in this study are systematically 15–30% lower than IPCC default values (e.g., coal: 18.5 kg/GJ measured vs. 25.9–36.0 kg/GJ IPCC; biomass: 2.1–2.5 kg/GJ measured vs. 23.1–32.0 kg/GJ IPCC [15]). This difference reflects fundamental methodological distinctions: IPCC Tier 1 factors assume 100% conversion of fuel carbon to CO_2_ (complete oxidation), providing conservative estimates suitable for national greenhouse gas inventories [25]. In contrast, our experimental measurements capture actual emissions under realistic combustion conditions where incomplete combustion produces CO alongside CO_2_ (5–15% of carbon emissions), and carbon retention occurs in char and ash residues. Industrial emission monitoring typically yields values intermediate between laboratory measurements and IPCC defaults due to longer residence times and higher temperatures in full-scale kilns. For cement industry applications, measured emission factors provide more accurate estimates for fuel comparison and operational decision-making, while IPCC Tier 1 factors remain appropriate for regulatory compliance calculations unless facility-specific data are available [15,16,25].

## 4. Discussion

The utilization of alternative fuels in cement production has emerged as a promising and environmentally sustainable approach to reducing coal dependency. Given that many alternative fuels demonstrate lower CO_2_ emission factors, their adoption can substantially reduce the industry’s carbon footprint. Substituting coal with these alternative fuels not only reduces greenhouse gas emissions but also promotes resource efficiency and waste management by repurposing materials that would otherwise require disposal.

The CO emission factors (kg/GJ) for the tested samples followed trends similar to those observed for total mass-based CO emissions. Most alternative fuels exhibited lower CO emission factors than coal, highlighting their potential in cleaner combustion applications. TDF represented the exception, producing 2.8 times higher CO emissions than coal, with higher CO emission factors likely attributable to its elevated fixed carbon content and reduced volatile matter [9]. In industrial applications such as those employing cement kilns, where high temperatures and extended residence times promote complete combustion, alternative fuels can be effectively utilized to achieve cleaner and more efficient burning. However, to further reduce CO emissions, optimization of combustion parameters—particularly air-to-fuel ratios—is essential. Increasing airflow or providing excess air enhances oxygen availability during combustion, promoting complete carbon oxidation to CO_2_ instead of CO. This approach is particularly important for fuels with a high fixed carbon content or reduced volatile matter, such as TDF, which tend to produce elevated CO levels under suboptimal combustion conditions.

The NO_x_ emission factors (kg/GJ) for alternative fuels were generally lower than those for coal, with the exception of IHW and TDF. This highlights the potential of many alternative fuels to serve as cleaner alternatives to coal, offering significant environmental benefits through reduced nitrogen oxide emissions during combustion. To maximize these benefits, careful control of fuel injection and combustion conditions is essential to further minimize NO_x_ formation. Additionally, blending various alternative fuels can promote more stable combustion, enhance fuel efficiency, and further reduce NO_x_ emissions, ultimately supporting more sustainable and environmentally friendly cement production processes.

The SO_2_ emission factor (kg/GJ) for TDF exceeded that of all other tested samples. Among the analyzed fuels, TDF consistently demonstrated the highest sulfur oxide emissions when normalized to energy content. Since SO_2_ emission reduction is critical for environmental protection, blending TDF with other fuels represents an effective strategy to reduce overall sulfur output during combustion.

The emission reductions achieved with alternative fuels translate to meaningful environmental and health benefits. Lower CO_2_ emissions (45% reduction for biomass-derived fuels) directly contribute to climate change mitigation, reducing long-term environmental and social impacts. Negligible SO_2_ emissions from most alternative fuels (except TDF) reduce acid rain formation, ecosystem acidification, and respiratory health risks associated with sulfur dioxide exposure. Reduced NO_x_ emissions from low-nitrogen biomass fuels decrease ground-level ozone formation (NO_x_ being a key ozone precursor) and associated respiratory diseases. However, elevated CO emissions from certain fuels (particularly TDF: 2.8× higher than coal) warrant attention due to carbon monoxide’s acute toxicity and role as an air quality pollutant. Our previous TGA-MS analysis [10] confirmed the absence of hazardous chlorinated compounds, dioxins, and furans from all tested fuels, indicating favorable toxicological profiles compared to uncontrolled waste combustion. Comprehensive health impact assessment would require atmospheric dispersion modeling, population exposure analysis, and epidemiological risk evaluation to fully quantify the public health implications of alternative fuel adoption in cement production.

Regarding compliance with emission standards, it is important to note that regulatory limits vary by jurisdiction and are typically expressed as concentration limits (mg/Nm^3^) at reference oxygen content (e.g., 10% O_2_) for continuous operation, whereas this study reports emission factors (kg/kg fuel or kg/GJ) from batch combustion tests. Nevertheless, the emission characteristics observed provide insight into compliance potential. Typical cement industry emission limits include NO_x_ < 200–400 mg/Nm^3^ (EU Industrial Emissions Directive), SO_2_ < 50–200 mg/Nm^3^, and CO < 500–1000 mg/Nm^3^. The low NO_x_ emissions from biomass fuels and negligible SO_2_ from most alternative fuels suggest favorable compliance margins for these pollutants. However, elevated CO from TDF and IHW indicates that these fuels may require combustion optimization or substitution rate limitations to maintain CO compliance, particularly under stringent regulatory scenarios. Industrial implementation should include continuous emission monitoring to verify compliance under site-specific conditions, recognizing that full-scale kiln performance may differ from laboratory results due to differences in temperature profiles, residence times, and combustion dynamics. The emission factors established here provide valuable guidance for preliminary compliance assessment and fuel screening.

It is important to note that while this study quantified major combustion products (CO_2_, CO, NO_x_, SO_2_), alternative fuel combustion may also produce trace quantities of other climate-active species including methane (CH_4_) and nitrous oxide (N_2_O). Under the oxidizing conditions employed here (λ = 1.5, 850 °C), CH_4_ formation is expected to be minimal (<0.1% of carbon emissions) due to rapid oxidation of any methane generated during pyrolysis. N_2_O emissions, which were not measured in this study, typically represent 1–10% of total NO_x_ emissions by mass but contribute disproportionately to greenhouse gas impacts due to high global warming potential (GWP_100_ = 265–298) [15]. Based on literature values for similar combustion conditions, estimated N_2_O emissions would range from <0.01 g/kg for low-nitrogen biomass to 0.1–0.5 g/kg for nitrogen-rich materials, contributing 1–5% to total CO_2_-equivalent emissions. Comprehensive climate impact assessments should incorporate these trace greenhouse gases alongside the dominant CO_2_ emissions.

IPCC emission factors are based on the theoretical complete oxidation of carbon to CO_2_, assuming 100% carbon conversion during combustion. In contrast, this study employed real laboratory measurements under simulated industrial conditions involving incomplete carbon oxidation, with some carbon converting to CO—a common, real-world byproduct of incomplete combustion that is difficult to eliminate completely—rather than CO_2_. As a result, the measured emission factors were consistently 15–30% lower than the theoretical IPCC values. This demonstrates that real-time experimental measurements provide more precise and industrially relevant emission data than standardized theoretical calculations, as they reflect actual combustion behavior under realistic operating conditions rather than idealized complete oxidation scenarios.

The emission factors established in this laboratory study provide essential baseline data for alternative fuel evaluation and comparison. However, translation of these results to industrial cement production requires consideration of scale-dependent factors. Industrial cement kilns operate with significantly different conditions including: higher temperatures in the burning zone (1400–1800 °C vs. 850 °C studied), shorter residence times in precalciner zones (3–5 s vs. 60–960 s observed), more complex gas-solid interactions with clinker bed and raw meal, turbulent mixing patterns in large-scale combustors, and continuous rather than batch fuel feeding [7,8].

Based on literature data comparing laboratory and industrial combustion systems, several systematic differences emerge. CO_2_ emission factors show relatively good agreement across scales (typically ±15–30% variation), as CO_2_ production is fundamentally determined by fuel carbon content and overall combustion stoichiometry [25,26]. Industrial measurements for similar coal combustion typically yield 18–20 kg CO_2_/GJ [15], consistent with our measured value of 19.4 kg CO_2_/GJ. However, combustion-sensitive species exhibit larger scale-dependent variations: CO emissions in industrial kilns may be 20–50% lower than laboratory batch tests due to enhanced mixing and longer high-temperature residence times that promote complete CO → CO_2_ oxidation [7,30]. Conversely, NO_x_ emissions can be 30–60% higher in industrial systems due to elevated peak flame temperatures (>1600 °C) that accelerate thermal NO_x_ formation, despite lower fuel-nitrogen conversion at moderate precalciner temperatures [5,8].

The controlled laboratory conditions employed in this study enable isolation of fuel-specific combustion behavior under reproducible conditions, providing fundamental emission data for fuel screening, comparison, and computational model validation. The carbon mass balance closure (±8%) and consistency with literature emission ranges confirm that the measurements capture essential combustion characteristics. Nevertheless, industrial validation through in-kiln measurements represents an essential next step, and such work is currently underway as part of our ongoing research program to confirm the applicability of these laboratory findings to full-scale cement production and to quantify scale-dependent emission variations for the specific alternative fuels characterized here.

The correlation between fuel composition and emission profiles provides valuable insights for fuel selection strategies. Materials with a higher volatile matter content and lower fixed carbon fractions (SD, PNS) demonstrated faster and more complete combustion, resulting in shorter combustion durations and lower emission factors. In contrast, fuels with complex chemical compositions and a higher fixed carbon content (TDF, IHW) required longer combustion times and produced elevated emissions of certain species. The correlation between fuel nitrogen content and NO_x_ formation confirms the importance of elemental composition in predicting combustion behavior, while the negligible SO_2_ emissions from most alternative fuels (except TDF) represent a clear environmental advantage over conventional fuels.

While this study focused on emission characterization, the heating values determined in our previous work [9] provide context for thermal efficiency implications. Based on literature data for cement production, specific heat consumption ranges from 3.0–3.5 GJ/t clinker for modern coal-fired kilns operating at high efficiency [5,57]. Alternative fuels with heating values comparable to or exceeding coal (TDF: 36.5 MJ/kg; PW: 29.0 MJ/kg; IHW: 34.0 MJ/kg) can maintain thermal efficiency with minimal adjustment to feed rates, potentially reducing specific heat consumption by 5–10% when substituting for lower-grade coals [7]. Conversely, lower-energy biomass fuels (SD: 16.9 MJ/kg; PNS: 18.0 MJ/kg) require proportionally higher mass feed rates—approximately 1.5–2.0 times the coal mass for equivalent thermal input—which may increase specific heat consumption by 0.2–0.4 GJ/t clinker at substitution rates above 30% (thermal basis) depending on moisture content and system configuration [57]. For example, replacing 20% of coal energy with dry sawdust (LHV: 16.9 MJ/kg, moisture: 8%) would theoretically increase clinker-specific heat consumption by approximately 0.1–0.15 GJ/t, while the same substitution with TDF (LHV: 36.5 MJ/kg, moisture: 1%) might reduce it by 0.05–0.10 GJ/t due to higher energy density. These estimates are indicative and highly dependent on kiln-specific parameters including preheater efficiency, fuel injection location, particle size distribution, and combustion air management. Industrial implementation requires integrated assessment balancing emission reductions (quantified in this study), thermal efficiency impacts, fuel costs, and operational considerations. Comprehensive heat balance modeling and industrial-scale validation represent important future work to optimize alternative fuel utilization strategies for specific cement production facilities.

From an economic perspective, the adoption of alternative fuels in cement production is driven by multiple factors beyond preprocessing costs. While size reduction and quality control require energy inputs (typically 15–50 kWh/t based on literature values), waste management economics often favor cement plants as waste receivers rather than generators [18,19]. Combined with avoided fossil fuel procurement costs and potential carbon credit benefits in regulated markets, alternative fuels frequently offer economic advantages alongside the emission reductions demonstrated in this study. However, comprehensive techno-economic assessment incorporating capital investments, operational modifications, quality assurance systems, and jurisdiction-specific regulatory frameworks represents important future work to fully quantify the business case for alternative fuel implementation in diverse regional contexts.

To optimize combustion performance and minimize emissions, several practical strategies can be implemented based on the characteristics observed in this study. For fuels exhibiting elevated CO emissions (particularly TDF), increasing the excess air ratio from λ = 1.5 to 1.7–2.0 provides additional oxygen to promote complete CO → CO_2_ conversion, although this must be balanced against potential flame temperature reduction and NO_x_ formation. Staged combustion—employing fuel-rich primary zones (λ = 1.2) to minimize thermal NO_x_, followed by secondary air injection to achieve overall λ = 1.6–1.8—can simultaneously reduce both CO and NO_x_ emissions [54]. Particle size reduction to <5 mm improves oxygen penetration into fuel particles and reduces diffusion limitations that promote incomplete combustion.

Strategic co-firing of multiple alternative fuels represents another potential approach to balance emission profiles, energy density, and operational requirements. The diverse fuel characteristics quantified in this study—ranging from high-volatile, low-nitrogen biomass (SD, PNS) to high-energy, nitrogen-rich synthetic materials (TDF, IHW)—suggest that carefully designed fuel combinations could leverage complementary properties to optimize overall performance. For example, co-firing high-volatile biomass with fixed-carbon-rich materials could stabilize combustion rates and reduce emission variability. However, fuel blending involves complex interactions during co-combustion that cannot be reliably predicted from individual fuel data alone. Synergistic effects (enhanced reactivity, improved burnout) or antagonistic effects (inhibited ignition, increased soot formation) may occur depending on mixing quality, volatile matter interactions, and ash chemistry. Systematic experimental investigation of specific fuel blends under controlled conditions, followed by industrial-scale validation, is necessary to quantify actual co-firing performance and emission profiles. Such blending optimization studies represent valuable future work that would build upon the single-fuel emission factors established here.

Temperature optimization within kiln operational constraints and ensuring adequate residence time (>5 s for complex fuels like TDF) further promote combustion completeness. These operational adjustments, guided by real-time emission monitoring, enable cement plants to maximize the environmental and economic benefits of alternative fuel utilization.

Industrial implementation of alternative fuels must consider material compatibility beyond emission profiles. Literature data indicate fuel-specific degradation mechanisms: (1) Refractory wear: high-chlorine fuels (> 0.5 wt% Cl) can reduce refractory service life by 30–50% through alkali-chloride attack on magnesia-spinel bricks, decreasing typical burning zone lifetimes from 8–12 months (coal) to 4–8 months [58]. (2) Metallic corrosion: chlorine- and sulfur-rich fuels increase corrosion rates by 2–15× for steel components exposed to acidic combustion gases, with preheater equipment particularly vulnerable [59]. (3) Abrasive wear: high-ash fuels (>15%) accelerate erosion of gas-solid contact surfaces and promote sticky deposit formation in preheaters [60].

Among the fuels studied here, elemental analysis [9] suggests varying degradation potential: biomass (SD, PNS) shows low corrosion risk (Cl < 0.05%, ash 1.4–4.5%); high-ash materials (WBW: 53%, ASR: 27%) may increase wear; TDF’s sulfur content (0.14%) warrants monitoring; plastic-derived fuels require assessment for trace chlorine. Comprehensive material compatibility evaluation—including ash analysis, elevated-temperature corrosion testing, and industrial monitoring of refractory consumption—represents essential complementary work for site-specific alternative fuel implementation strategies.

The integration of alternative fuels into cement manufacturing presents a multifaceted environmental solution that extends beyond direct emission reductions. It supports waste management and resource efficiency while contributing to circular economy principles. Through strategic fuel selection, optimized combustion conditions, and appropriate blending strategies, cement plants can achieve significant emission reductions while maintaining operational efficiency and compliance with evolving environmental regulations.

## 5. Conclusions

This study confirms the environmental viability of alternative fuels as sustainable substitutes for coal in cement production. Its key findings are outlined below.

CO_2_ Emissions: Alternative fuels consistently produced lower CO_2_ emission factors than coal, with biomass-derived materials (SD and PNS) achieving the greatest reductions of 45% and 38%, respectively. All measured emission factors were 15–30% lower than theoretical IPCC values, highlighting the importance of experimental measurements over theoretical estimates. Given their strong climate benefits, biomass-based fuels such as SD and PNS should be prioritized where greenhouse gas mitigation is the main objective.

CO Emissions: Most alternative fuels generated lower CO emissions than coal, except TDF, which exhibited emissions 2.8 times higher. The shorter combustion durations of volatile-rich fuels indicate enhanced reactivity and efficient thermal decomposition. However, fuels with elevated CO emissions, such as TDF, require combustion optimization—through increased excess air, particle size reduction, or co-firing—to minimize incomplete combustion penalties.

NO_x_ Emissions: Alternative fuels generally produced lower NO_x_ emissions than coal due to their lower nitrogen content, with IHW and TDF being exceptions because of their elevated nitrogen concentrations. Low-nitrogen fuels, particularly biomass (<0.5% N), are preferable in contexts with stringent air quality regulations, whereas high-nitrogen fuels (TDF, IHW) may require additional NO_x_ control technologies to ensure compliance.

SO_2_ Emissions: SO_2_ emissions were negligible for all alternative fuels except TDF (0.14% sulfur), demonstrating a clear environmental advantage over conventional fuels. Materials with negligible sulfur content (<0.01%) effectively prevent SO_2_ emissions, while sulfur-bearing fuels like TDF require assessment of their SO_2_ contribution and potential sulfur cycling issues within the kiln system.

Progressive substitution of coal with biomass-derived alternative fuels (achieving 45% CO_2_ reduction per unit energy) could reduce global cement industry emissions by approximately 9–11% annually if substitution rates increase from 20% to 50%. However, realizing these benefits requires sustainable feedstock management, optimized collection and preprocessing logistics, and integration with complementary decarbonization technologies. Comprehensive lifecycle assessments incorporating temporal and systemic factors represent important future research directions.

Maintaining adequate fuel energy density (>20 MJ/kg preferred) is essential to avoid excessive feed rates and thermal efficiency penalties; alternatively, low-energy fuels can be blended with high-energy materials. Ash content and mineralogy should be evaluated to ensure compatibility with clinker chemistry and to prevent operational issues such as buildups or blockages. Furthermore, locally available waste streams should be prioritized to minimize transportation costs and environmental footprint, while considering both fuel cost and potential tipping fee revenues from waste disposal services.

The emission factors generated in this study provide cement manufacturers with essential data for environmental impact assessments, regulatory compliance, and informed decision-making regarding sustainable fuel adoption. Collectively, these findings support the technical and environmental feasibility of transitioning from coal to alternative fuels as a viable pathway toward more sustainable cement production processes.

Finally, this study establishes baseline emission factors under controlled laboratory conditions. Several research directions would advance industrial implementation: (1) Multi-temperature studies: investigating combustion and emissions across multiple temperatures (650 °C, 850 °C, 1050 °C) would elucidate temperature-dependent emission profiles and optimize injection strategies for different kiln zones. (2) Industrial-scale validation: full-scale cement kiln trials with continuous emission monitoring are essential to validate laboratory findings under actual production conditions, accounting for complex aerodynamics, clinker bed interactions, and operational variability. (3) Combustion optimization: systematic evaluation of operational parameters (excess air ratio, particle size, residence time) would identify optimal conditions for minimizing emissions while maintaining thermal efficiency. (4) Fuel blending studies: the diverse characteristics of alternative fuels quantified here (volatile matter, nitrogen content, heating value, combustion kinetics) suggest that strategic co-firing combinations could optimize emission profiles and operational stability. However, fuel blending involves complex co-combustion interactions that require systematic experimental investigation to quantify synergistic or antagonistic effects, optimal mixing ratios, and impacts on clinker chemistry. Such studies would build upon the single-fuel emission factors established in this work. (5) Long-term operational studies: extended trials (months to years) would assess impacts on equipment wear, refractory degradation, kiln stability, clinker quality, and maintenance requirements. (6) Life cycle assessment: comprehensive LCA incorporating fuel collection, preprocessing, transportation, combustion, and end-of-life considerations would provide complete environmental footprinting beyond direct combustion emissions. (7) Economic analysis: techno-economic modeling including capital costs, operational expenses, carbon pricing, and waste disposal revenues would inform investment decisions. (8) Trace species characterization: measurement of CH_4_, N_2_O, and organic pollutants would complete the greenhouse gas inventory and address air toxics concerns.

## Figures and Tables

**Table 1:** Literature data on the influence of excess air ratio on CO and NO_x_ emissions during solid fuel combustion.

Fuel Type	λ Range	CO Trend *	NO_x_ Trend *	References
Bituminous coal	1.2–1.8	Decreases 60–80% as λ increases from 1.2 to 1.5; minimal change above λ = 1.5	Increases 20–40% from λ = 1.3 to 1.8 due to enhanced oxidation conditions	[20,22]
Wood biomass	1.3–2.0	Sharp decrease below λ = 1.5 (incomplete combustion); stable above λ = 1.5	Low overall (<50 ppm); slight increase (10–20%) at λ > 1.7	[20,24]
Tire-derived fuel	1.4–1.8	High baseline CO; decreases 40–50% from λ = 1.4 to 1.6; modest further reduction at higher λ	Increases linearly ~ 15–25% per 0.1 λ increment due to fuel-N oxidation	[21,23]
Mixed waste	1.3–2.0	Highly variable; optimal range λ = 1.5–1.7 for CO minimization	Moderate sensitivity; increases 30–50% from λ = 1.5 to 2.0	[20,21,24]

* Trends are approximate and system-dependent, varying with temperature, residence time, fuel properties, and combustion configuration.

**Table 2:** Emission factors expressed per unit of fuel mass (kg/kg) and energy content (kg/GJ).

Sample	CO_2_		CO		NO_x_		SO_2_	
	$\frac{kg}{kg}$	$\frac{kg}{GJ}$	$\frac{kg}{kg}$	$\frac{kg}{GJ}$	$\frac{kg}{kg}$	$\frac{kg}{GJ}$	$\frac{kg}{kg}$	$\frac{kg}{GJ}$
SD	0.0400 ± 0.0022	2.3669 ± 0.0478	0.0082 ± 0.0005	0.4852 ± 0.0098	*	0.0006 ± (*)	-	-
PNS	0.0507 ± 0.0011	2.8167 ± 0.0569	0.0219 ± 0.0001	1.2167 ± 0.0246	*	0.0022 ± (*)	-	-
WBW	0.0468 ± 0.0010	4.0696 ± 0.0822	0.0190 ± 0.0004	1.6522 ± 0.0334	0.0001 ± (*)	0.0087 ± 0.0002	-	-
IHW	0.3476 ± 0.0071	10.2235 ± 0.2065	0.0606 ± 0.0012	1.7824 ± 0.0360	0.0007 ± (*)	0.0191 ± 0.0004	-	-
TDF	0.2319 ± 0.0048	6.3534 ± 0.1283	0.1112 ± 0.0023	3.0466 ± 0.0615	0.0018 ± (*)	0.0496 ± 0.0010	0.0016 ± (*)	0.0430 ± (*)
PW	0.2405 ± 0.0049	8.2931 ± 0.1675	0.01812 ± 0.0004	0.6248 ± 0.0126	0.0002 ± (*)	0.0062 ± 0.0001	-	-
ASR	0.0838 ± 0.0017	3.8091 ± 0.0769	0.0079 ± 0.0002	0.3591 ± 0.0073	0.0002 ± (*)	0.0082 ± 0.0002	-	-
Coal	0.5406 ± 0.0112	19.4460 ± 0.3928	0.0715 ± 0.0015	2.5719 ± 0.0520	0.0005 ± (*)	0.0191 ± 0.0004	-	-

* and (*) indicate values < 0.0001 kg/kg; “-” indicates not detected.

## Data Availability

The original contributions presented in this study are included in the article. Further inquiries can be directed to the corresponding authors.
